# Enhanced temporal complexity of EEG signals in older individuals with high cognitive functions

**DOI:** 10.3389/fnins.2022.878495

**Published:** 2022-09-21

**Authors:** Yuta Iinuma, Sou Nobukawa, Kimiko Mizukami, Megumi Kawaguchi, Masato Higashima, Yuji Tanaka, Teruya Yamanishi, Tetsuya Takahashi

**Affiliations:** ^1^Graduate School of Information and Computer Science, Chiba Institute of Technology, Narashino, Japan; ^2^Department of Preventive Intervention for Psychiatric Disorders, National Center of Neurology and Psychiatry, National Institute of Mental Health, Tokyo, Japan; ^3^Faculty of Medicine, Institute of Medical, Pharmaceutical and Health Sciences, Kanazawa University, Kanazawa, Japan; ^4^Department of Nursing, Faculty of Medical Sciences, University of Fukui, Yoshida, Japan; ^5^Medical Corporation Aokikai Seiwa Hospital, Kanazawa, Japan; ^6^Akashi Mind Hospital, Akashi, Japan; ^7^Faculty of Education, Osaka Seikei University, Osaka, Japan; ^8^Research Center for Child Mental Development, Kanazawa University, Kanazawa, Japan; ^9^Department of Neuropsychiatry, Faculty of Medical Sciences, University of Fukui, Yoshida, Japan; ^10^Uozu Shinkei Sanatorium, Uozu, Japan

**Keywords:** cognitive function, complexity analysis, EEG, multiscale entropy analysis, Five-Cog

## Abstract

Recent studies suggest that the maintenance of cognitive function in the later life of older people is an essential factor contributing to mental wellbeing and physical health. Particularly, the risk of depression, sleep disorders, and Alzheimer's disease significantly increases in patients with mild cognitive impairment. To develop early treatment and prevention strategies for cognitive decline, it is necessary to individually identify the current state of cognitive function since the progression of cognitive decline varies among individuals. Therefore, the development of biomarkers that allow easier measurement of cognitive function in older individuals is relevant for hyperaged societies. One of the methods used to estimate cognitive function focuses on the temporal complexity of electroencephalography (EEG) signals. The characteristics of temporal complexity depend on the time scale, which reflects the range of neuron functional interactions. To capture the dynamics, composed of multiple time scales, multiscale entropy (MSE) analysis is effective for comprehensively assessing the neural activity underlying cognitive function in the brain. Thus, we hypothesized that EEG complexity analysis could serve to assess a wide range of cognitive functions in older adults. To validate our hypothesis, we divided older participants into two groups based on their cognitive function test scores: a high cognitive function group and a low cognitive function group, and applied MSE analysis to the measured EEG data of all participants. The results of the repeated-measures analysis of covariance using age and sex as a covariate in the MSE profile showed a significant difference between the high and low cognitive function groups (*F* = 10.18, *p* = 0.003) and the interaction of the group × electrodes (*F* = 3.93, *p* = 0.002). Subsequently, the results of the *post-hoc* *t*-test showed high complexity on a slower time scale in the frontal, parietal, and temporal lobes in the high cognitive function group. This high complexity on a slow time scale reflects the activation of long-distance neural interactions among various brain regions to achieve high cognitive functions. This finding could facilitate the development of a tool for diagnosis of cognitive decline in older individuals.

## 1. Introduction

In a super-aging society, cognitive decline in older adults is a pressing issue (Gauthier et al., 2006). In particular, maintaining a high cognitive function is important for the mental wellbeing of older individuals (Cohen, 2006; McFadden and Basting, 2010; Ueno et al., 2015). Therefore, maintaining high cognitive function in later life is essential to optimize mental wellbeing and physical health (Hendrie et al., 2006; Depp et al., 2011).

In patients with mild cognitive impairment (MCI), the risk of depression, sleep disorders, and dementia increases significantly (Petersen et al., 1999; Guarnieri and Sorbi, 2015; Snowden et al., 2015). Additionally, the rate of conversion from MCI to dementia is high and a return to a healthy state from MCI can be challenging (Gabryelewicz et al., 2007; Farias et al., 2009; Marcos et al., 2016). Therefore, preventive interventions for MCI are important (Roberts and Knopman, 2013). Furthermore, increases personal and social burdens such as medical insurance and patient care are necessary to deal with MCI and dementia. If both the onset and progression of cognitive decline could be delayed through early intervention, the number of people requiring a high level of care would decrease (Brookmeyer et al., 2007). Advances in treatment and prevention strategies, which lead to a delay in cognitive decline, can significantly reduce personal and social burdens (Brookmeyer et al., 2007). Therefore, approaches toward maintaining high cognitive function in older individuals are desired (Gates et al., 2019). However, individual differences in the symptoms of cognitive decline depend on various environmental factors, such as lifestyle and other personal factors (Stern, 2012; Gates et al., 2019). In such scenario, with individually varying cognitive decline progression, tailor-made support programs are required to efficiently maintain a high cognitive function (reviewed in Beattie et al., 2002).

One of the essential factors in establishing tailor-made support programs is estimating the current state of cognitive function individually. Currently, cognitive function tests, such as the Mini Mental State Examination (MMSE) and the Montreal Cognitive Assessment, are widely used in clinical practice (Smith et al., 2007; Wong et al., 2015). These tests impose a considerable burden in older individuals and medical resources since they are conducted multiple times within a certain period; each test is face-to-face and requires a long time (Arevalo-Rodriguez et al., 2015; Wong et al., 2015). Additionally, the diagnosis of cognitive decline requires analysis using cognitive indicators combining multiple biomarkers in longitudinal measures (Aisen et al., 2011). Therefore, the development of new biomarkers that can objectively and quantitatively assess cognitive function in older adults is crucial.

In recent years, studies based on the temporal dynamics of neural activity using electroencephalography (EEG), magnetoencephalography (MEG), and functional magnetic resonance imaging (fMRI) have revealed neural activity across neural networks in the brain (Kuller et al., 1998; Camp et al., 1999; Debener et al., 2006; Nobukawa et al., 2019a, 2020b). Among these neuroimaging methods, EEG is cost-effective, widely available, and noninvasive, making it suitable for clinical applications (Vecchio et al., 2013; Kulkarni, 2018). An estimation of cognitive decline by EEG demonstrated that EEG's power spectrum was associated with reduced performance in multiple advanced cognitive function areas (Van der Hiele et al., 2007). In addition, Jelic et al. (2000) reported that the indices associated with alpha and theta relative powers in the left temporal lobe can significantly distinguish MCI patients with and without progressive cognitive decline. In another study examining the relationship between the power spectrum and cognitive decline, Elmståhl and Rosén (1997) revealed that low beta activity in the EEG reflects cognitive decline in older individuals. Therefore, frequency band-specific EEG activity can estimate cognitive decline by capturing the neural activity of extensive neural networks in the brain. However, the power of brain activity in EEG signals mainly reflects local brain activity. The integration of information from various brain neural networks plays an important role in optimal brain function (Varela et al., 2001; Buzsaki and Draguhn, 2004; Fries, 2005; Hutchison et al., 2013; Koutsoukos et al., 2015). Therefore, it is important to assess not only these local brain activities, but also the interactions among global neural networks.

One of the methods reflecting the interaction of neural activity between brain regions focuses on the functional connectivity among neural networks (Mišić et al., 2011; Tobe and Nobukawa, 2021). Functional connectivity reflects the integration of brain information processing between brain regions as the mutual interaction of neural activity (Varela et al., 2001; Fell and Axmacher, 2011; Hardmeier et al., 2014); especially, methods focusing on network connectivity have been widely used to reflect brain activity through age-related cognitive decline and the degree of cognitive function in older individuals (Ferreira and Busatto, 2013; Damoiseaux, 2017; Nobukawa et al., 2020b). However, in functional connectivity, the interaction between many areas, typified as cognitive function, needs to be assessed in terms of whole-brain network characteristics as network topology (e.g., node degree and centrality) (Zeng et al., 2015; Makarov et al., 2018). Therefore, to assess the topological features of the whole-brain network, a highly dense EEG is needed (Mišić et al., 2011; Hardmeier et al., 2014), which restricts clinical availability.

Another method for evaluating the neural interactions underlying cognitive processes focuses on the temporal complexity of neural activity (Takahashi, 2013; Ueno et al., 2015; Ando et al., 2021; Iinuma et al., 2022). These complex temporal variabilities in brain activity play an important role in perception and mental and behavioral processes and are mechanisms of stochastic resonance and stochastic facilitation (McDonnell and Ward, 2011; Garrett et al., 2013b; Takahashi, 2013; Yang and Tsai, 2013; Nobukawa et al., 2020b). In particular, in the study of cognitive functional changes related to age and the pathology of cognitive decline, the application of complexity analysis, such as multifractal analysis, the correlation dimension, and Lyapunov exponent to EEG, has been widely used to quantitatively characterize complexity (Lee et al., 2000; Takahashi et al., 2009; Ueno et al., 2015; Zorick et al., 2020; Ando et al., 2021; Ma et al., 2021). Furthermore, the complexity obtained from local brain regions reflects the topological characteristics of whole-brain functional connectivity (Mišić et al., 2011; Ando et al., 2022). Therefore, complexity analysis with low-density EEG is suitable for developing biomarkers for evaluating cognitive decline in older individuals.

Moreover, the complexity of brain activity depends on the time scale and corresponds to interactions between brain regions (Wang, 2010). In particular, neural activity on fast time scales reflects local inter-regional neural activity, whereas neural activity on slow time scales reflects long-range neural activity (Wang et al., 2018). Thus, the dynamics of brain activity are composed of neural activity on multiple time scales (Fisher et al., 2004). In particular, the time scale dependent neural variability obtained from EEG has been shown to reflect the relationship between cognitive function, aging, and creativity (Takahashi et al., 2009, 2016; Ueno et al., 2015; Nobukawa et al., 2019a; Ando et al., 2022). Therefore, multiscale entropy (MSE) analysis has been widely used to quantify complexity and assess brain activity using multiple time scales (Costa et al., 2002; Takahashi, 2013). The application of MSE analysis to EEG was useful to capture changes in the complexity of EEG dynamics associated with creativity and aging (Takahashi et al., 2009; Ueno et al., 2015; Nobukawa et al., 2019a, 2020a; Ando et al., 2021).

Various approaches have been conducted in the past, concerning the estimation of cognitive function using EEG (Ueno et al., 2015; Nobukawa et al., 2019a, 2020a; Ando et al., 2021). However, previous studies on cognitive function and EEG complexity have focused on limited cognitive functions, such as disease-level cognitive decline, specific cognitive functions, and aging network alternation (Ueno et al., 2015; Nobukawa et al., 2019a, 2020a; Ando et al., 2021). A more comprehensive and detailed time scale dependent assessment of cognitive function and complexity has not been conducted so far. For preventive interventions in a hyperaging society, it is important to deal with these evaluations. Therefore, we hypothesized that focusing on the time scale dependent complexity of functional neural activity in the brain, captured by EEG, would allow us to evaluate a wide range of cognitive functions in older people. To validate this hypothesis, this study aimed to study the potential of EEG to reveal high and low cognitive function in order people. In this study, we divided healthy older people into two groups based on their high and low scores on cognitive function tests, and applied MSE analysis to the EEG data of each participant to derive EEG complexity on multiple time scales and detect the changes between the groups with high and low cognitive functions. In addition, to confirm the usefulness of MSE analysis in this study, we conducted power spectrum density analysis to confirm the advantage of the method.

## 2. Methods

### 2.1. Participants

We gathered 199 people form the communal society in the Eiheiji-cho. For comparison, we recruited 43 healthy older medication-free participants (age range: 65–85 years old), based on the following exclusion criteria: major medical or neurological conditions, history of alcohol or drug dependency, and internal diseases, including hypertension, hyperlipidemia, and diabetes mellitus. Based on these exclusion criteria, participants with relatively high cognitive function for their age were chosen in the communal society. In particular, no participants with MCI satisfying with mini mental state examination (MMSE) threshold (<24) (Trzepacz et al., 2015). To test cognitive function, we used the Five-Cognitive Functions (Five-Cog) test, developed by the International Psychogeriatric Association to detect cognitive decline in the older individuals (Miyamoto et al., 2009). This test consists of five items: “character position matching task” to measure attention, “category cued recall task” to measure memory, “clock drawing task” to measure visuospatial functional ability, “word recall task” to measure language ability, and “similar word task” to measure thinking ability (Fujii et al., 2021). The “character position matching task” is an attention-splitting task that requires attention switching and declines particularly at the stage of MCI. The “category cued recall task” is a task that indicates impaired episodic memory function at the stage of MCI. The “similar word task” can easily decline with MCI. In contrast, the visual component and the ability to draw out appropriate words do not decline much at the stage of MCI, but they do in Alzheimer's disease (AD). The “clock drawing task” and “word recall task” are tasks that indicates the decline in these abilities. In this study, the sum of the scores of all Five-Cog domains in each individual were used as total score. As criterion, considering the situation for restricting size of participants, the median of the distribution is appropriate to divide two groups with the same size. Therefore, the participants were divided into two groups based on the median of their total Five-Cog score: a high cognitive function group and a low cognitive function group. The basic characteristics of the participants in the two groups is shown in Table 1. The education level in the high cognitive group was significantly higher than in the low cognitive group. However, as the Five-Cog test scores were adjusted based on educational history, this difference does not affect our results. Additionally, there were no significant differences in blood pressure or BMI between groups. All participants provided informed consent before the beginning of the study. The study protocol was approved by the Ethics Committee of Fukui University. All procedures were conducted in accordance with the principles of the Declaration of Helsinki.

**Table 1:** Basic composition of the participants in high and low cognitive functioning groups based on Five-Cognitive Functions (Five-Cog) scores.

	**High cognitive group**	**Low cognitive group**	***p*-value**
Mean age [Standard deviation (sd)]	70 (4.30)	74.14 (5.22)	**0.007**
Mean education history [Standard deviation (sd)]	12.0 (1.57)	11.9 (2.45)	0.8745
Mean total score of Five Cog (sd)	168.27 (10.04)	142.52 (8.28)	**< 0.001**
Mean BMI values	23.636	23.4078	0.832
Mean blood pressure (Systolic)	139.900	137.182	0.599
Mean blood pressure (Diastolic)	80.150	78.546	0.652
Male/Female	6/16	7/14	0.665

The *p*-value of the group difference between the high and low cognitive function groups with *p* < 0.05 is indicated in bold characters.

### 2.2. EEG recordings

EEG data were recorded using a 21-channel system (EEG-1514, Nihon Kohden, Tokyo) at 19 electrode sites (Fp1, Fp2, F3, F4, C3, C4, P3, P4, O1, O2, F7, F8, T3, T4, T5, T6, Fz, Cz, and Pz), in accordance with the international 10-20 system, with the two ear lobes jointly forming the reference. The participants sat comfortably on chairs in an electrically shielded, soundproof, and dimmed room. During EEG recordings, they were in a state of wakefulness with their eyes closed for ≥3 min. The EEG signals were recorded with a sampling frequency of 500 Hz, a 1–60 Hz bandpass filter, and a time constant of 0.3 s. Since the bandpass-filtered data contained little line noise at 60 Hz, a notch filter was not applied. The electrode impedances were < 5 kΩ. Artifacts, including eye movements, blinks, and muscle activity, were manually excluded.

### 2.3. MSE analysis

**Figure 1:**
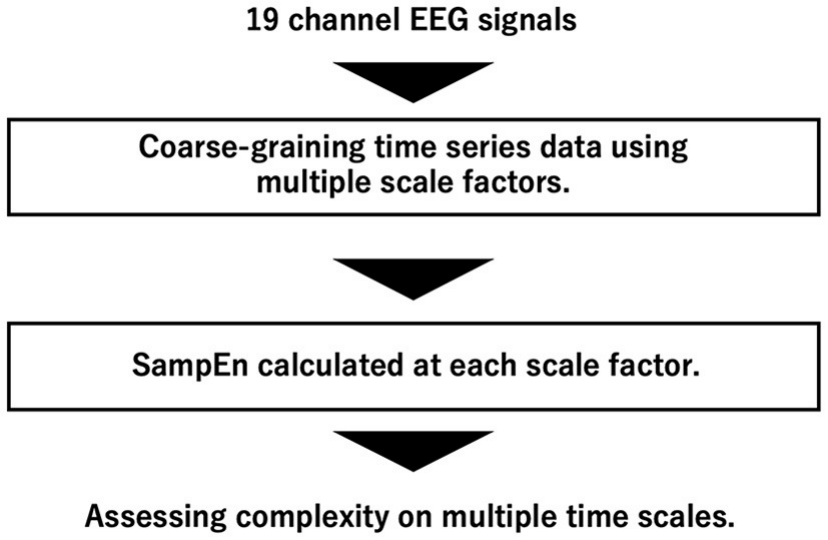
Overview of complexity analysis of EEG signals by multi-scale entropy (MSE) analysis.

An overview of complexity analysis of EEG signals by MSE analysis is shown in Figure 1. MSE analysis is a method for quantifying the complexity of time-series data at multiple time scales by coarse-graining (Costa et al., 2005).

First, compared to the original EEG time series, {*x*_1_, *x*_2_, ⋯, *x*_*N*_} were coarse-grained using the time scale factor (τ) with a non-overlapping window by


(1) $$\begin{array}{l} y_{j}^{\tau }=\left(\frac{1}{\tau }\right)\overset{j\tau }{\underset{i=\left(j-1\right)\tau +1}{\sum }}x_{i},\left(1\leq j\leq \frac{N}{\tau }\right) \end{array}$$

where {*y*_1_, *y*_2_, ⋯, *y*_*N*_} are the obtained coarse-grained signals. {*y*_1_, *y*_2_, ⋯, *y*_*N*_} were converted using the *Z*score.

Since the temporal complexity of {*y*_1_, *y*_2_, ⋯, *y*_*N*_}, sample entropy (SampEn) was defined by


(2) $$\begin{array}{l} S_{E}\left(m,r\right)=-\log\frac{U_{m+1}\left(r\right)}{U_{m}\left(r\right)}. \end{array}$$

*U*_*m*_(*r*) is the probability that $|y_{i}^{m}-y_{j}^{m}|<r\left(i\neq j,i,j=1,2,\cdots \;\right)$. $y_{i}^{m}$ is an *m*-dimensional vector, $y_{i}^{m}=\left\{y_{i},y_{i+1},\cdots \;,y_{i+m-1}\right\}$. τ (τ = 1, 2, ⋯) is the time scale. In this study, we set *m* = 2 and *r* = 0.2 (Costa et al., 2005).

The SampEn values obtained with smaller scale factors capture the temporal complexity arising from short-range interactions, whereas higher scale factors are associated with temporal complexity produced by long-range interactions (Ueno et al., 2015). Therefore, evaluating the complexity at multiple time scales provides a more comprehensive assessment of the complexity of EEG signals as the temporal-scale profile of inherent dynamics (Costa et al., 2005; Ueno et al., 2015; Nobukawa et al., 2019a,b, 2020a, 2021). In this study, SampEn derived from 40 scales was averaged and evaluated for each of the five scales, that is, averaged eight time scale ranges: 1–5 scale (5–25 ms), 6–10 scale (30–50 ms), 11–15 scale (55–75 ms), 16–20 scale (80–100 ms), 21–25 scale (105–125 ms), 26–30 scale (130–150 ms), 31–35 scale (155–175 ms), 36–40 scale (180–200 ms). In addition, as the number of epochs differs among individuals, SampEn is averaged across epochs (mean number of epochs among individuals: 12.12).

### 2.4. Power spectrum

In addition to the complexity analyzed by MSE analysis, we computed the power spectral density (PSD) of our EEG signals. In this analysis, we estimated PSD in dB/Hz using Welch's method with a Hanning window function with a width of 2.0 s.

### 2.5. Statistical analysis

For SampEn, repeated measures analysis of covariance (ANCOVA) with the groups (high cognitive function group vs. low cognitive function group) as between-subject factor, electrodes (19 electrodes from Fp1 to Pz) and time scales (8 scale ranges averaged per scale 5) as within-subject factors, and age and sex as the covariates was performed to test for group differences. The Greenhouse-Geisser adjustment and a α bilateral level of 0.05 were applied. The result of the ANCOVA was represented by the *F*-value based on the comparison of covariances within/between groups. The *post-hoc* *t*-test was used to assess the significant main effects of the group and per-electrode and per time scale interactions. Benjamini-Hochberg false discovery rate (FDR) correction was applied to the *t*-scores for multiple comparisons (*q* < 0.05) (152 *p*-values: 19 electrodes × 8 scale ranges). For the electrode-wise group comparisons of the PSDs, a *t*-test with an FDR correction was also used. As well as for MSE analysis, *q* < 0.05 was applied (1, 121 *p*-values: 59 frequency points [2–60 Hz; width of the frequency bin, 1.0 Hz] × 19 electrodes).

## 3. Results

### 3.1. MSE analysis

**Figure 2:**
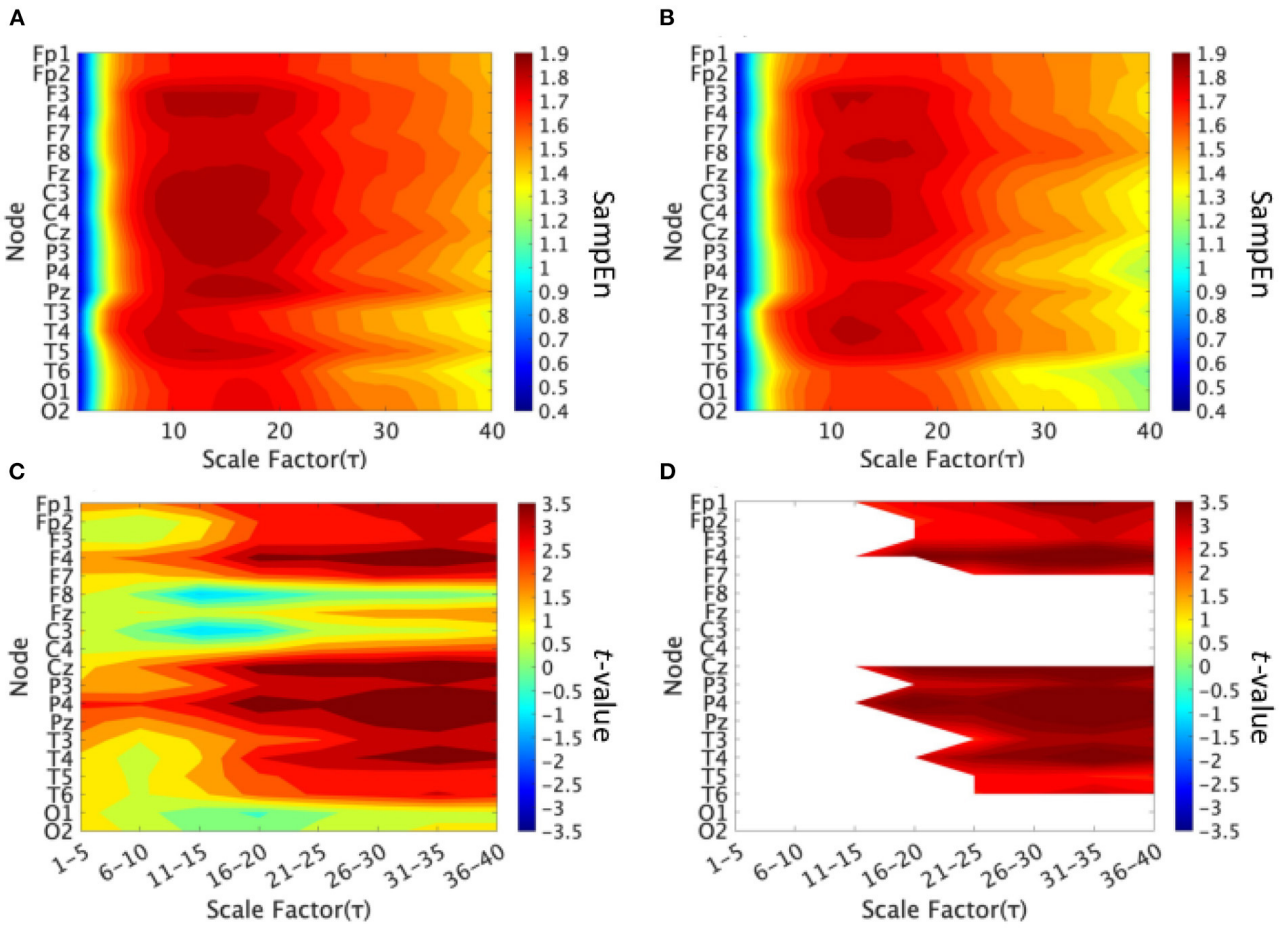
Multi-scale entropy analysis in the high and low cognitive function groups. The horizontal axis represents the temporal-scale factor τ. **(A)** Mean values of sample entropy (SampEn) from scale factors 1 (0.005 s) to 40 (0.2 s) in the high cognitive function group. **(B)** Mean values of SampEn from scale factors 1 (0.005 s) to 40 (0.2 s) in the low cognitive function group. **(C)** *t*-value between the high and low cognitive function groups. The warm (cold) color represents a higher (smaller) SampEn value for the high cognitive function group compared to that of the low cognitive function group. **(D)** The *t*-value satisfying the FDR correction criteria *q* < 0.05 corresponding to (*p* < 0.002). A significantly higher SampEn of the high cognitive function group was confirmed at slow time scale regions 11–40 (55–200 ms).

We performed an MSE analysis in the high and low cognitive function groups. Table 2 shows the repeated-measures ANCOVA results from the MSE analysis. A significant large main effect of SampEn group differences was confirmed as was a significant large interaction effect of group × nodes. As a *post-hoc* *t*-test, the mean SampEn values in the high and low cognitive function groups and the *t*-values between the high and low cognitive function groups are shown in Figure 2. The results showed that EEG complexity tended to be higher in the high cognitive function group than in the low cognitive function group in the slow time scale regions 11 to 40 (55–200 ms). In particular, 13 electrodes (Fp1, Fp2, F3, F4, P3, P4, F7, T3, T4, T5, T6, Cz, and Pz) passed the FDR correction, and the results confirmed site specificity from the frontal to the parietal and temporal lobes of the brain.

**Table 2:** High cognitive function group vs. low cognitive function group based on the repeated measure ANCOVA results *F*-value (*p*-value) and partial η^2^-value in multi scale entropy (MSE) analysis results, *F* and *p*-value with *p* < 0.05 are indicated by bold characters.

	***F*-value (*p*-value)**	**Partial η^2^**
**Group**	**10.181 (0.003)**	**0.207**
Group × scale	1.295 (0.279)	0.032
**Group × node**	**3.930 (0.002)**	**0.092**
Node × scale × group	1.150 (0.332)	0.029

### 3.2. Power spectrum analysis

**Figure 3:**
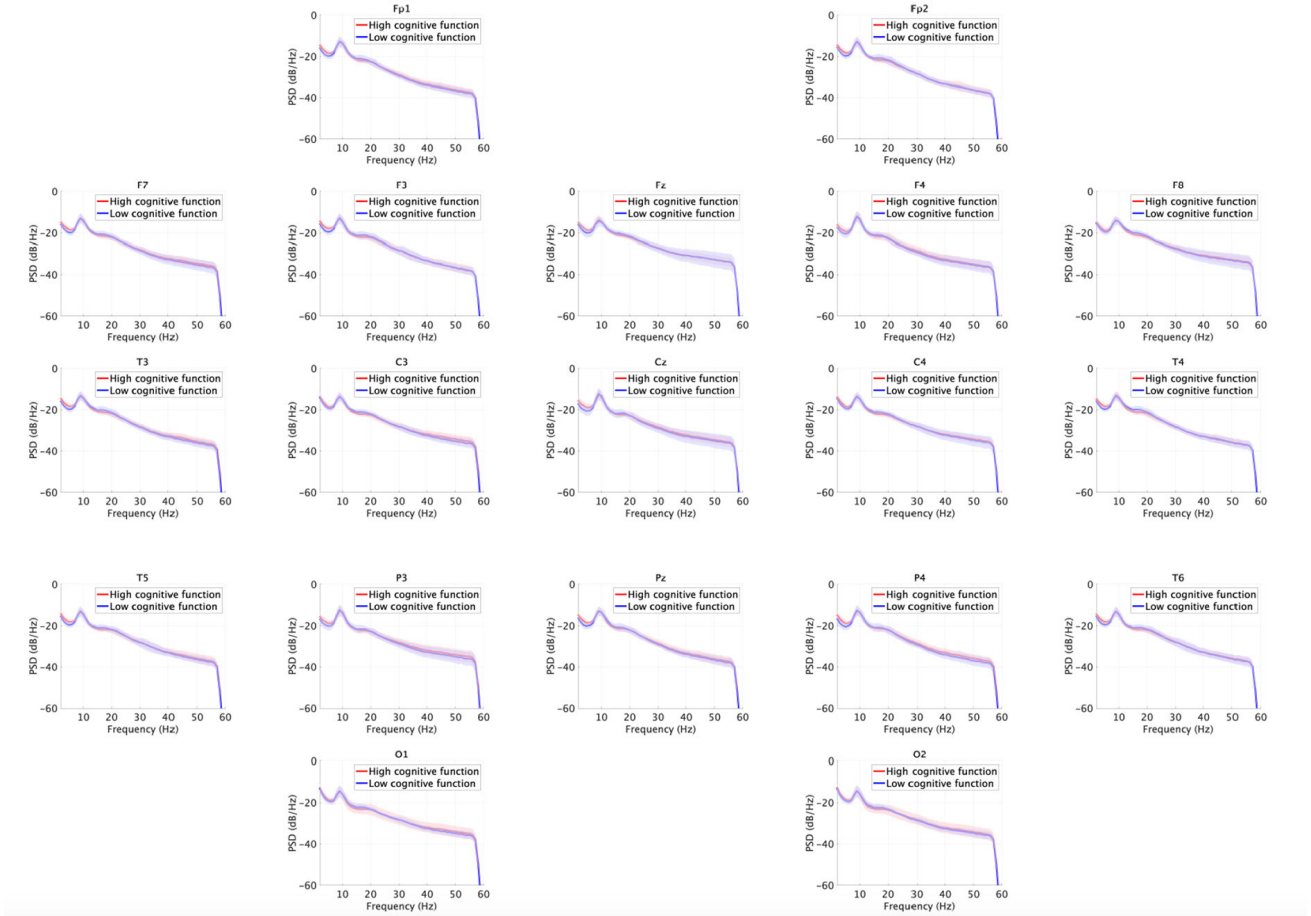
EEG power spectral density (PSD) for the high and low cognitive function groups. Solid lines and shaded areas indicate mean and standard deviation in each group. We found no significant differences after adjusting for false discovery rate (FDR) with *q* < 0.05.

Figure 3 shows the EEG PSD for the high and low cognitive function groups. In this power evaluation, we did not find significant differences after adjusting for FDR at *q* < 0.05.

## 4. Discussion and conclusion

To identify the early signs of cognitive decline in healthy older adults, we divided them into two groups, one with high cognitive function and the other with low cognitive function, based on their cognitive function test scores. The results revealed a significant difference in the complexity between the two groups, and higher EEG complexity in the high cognitive function group. High complexity was observed in the frontal, parietal, and temporal lobes, and the tendency for increasing complexity was observed on the slow time scale.

First, we considered the reasons for the high EEG complexity in the high cognitive function group. Cognitive functions emerge in neural networks connected by long-range brain regions; therefore, their related neural activity reflects long-range neuronal interactions (Tononi et al., 1998; Garrett et al., 2013a), which involve a high degree of information integration (Tononi et al., 1998). Several studies have reported that older people with low cognitive function exhibit less temporal complexity in their brain activity (Garrett et al., 2013a; Ishii et al., 2017). The results obtained in this study are highly congruent with previous findings. In addition, the time scale ranges reflecting cognitive function are not arbitrary but reflect the frequency-band specific functional networks (Miraglia et al., 2017; Nobukawa et al., 2020b). In this study, the increased complexity of dynamics on the 55–200 ms time scale corresponds to a frequency component of approximately 5–18 Hz, corresponding to the alpha and theta bands. Previously, Nobukawa et al. (2020b) revealed increased whole-brain functional connectivity of the alpha band in the high cognitive group. In addition, Vecchio et al. revealed decreased functional connectivity in the theta band in AD/MCI in comparison with healthy aging (Miraglia et al., 2017). This indicates that cognitive function is supported by the appropriate theta-band functional connectivity strength. Based on the above, the enhancement of whole-brain neural interactions, associated with increased functional connectivity in the alpha and theta bands and strongly related to high cognitive function, can increase the complexity of dynamics on the 55–200 ms time scale. This finding is also supported by previous findings on the correlation between functional connectivity strength and the complexity of neural activity (Sporns et al., 2007; Mišić et al., 2011; Ando et al., 2022).

Second, we considered the reasons for the high complexity of the frontal, parietal, and temporal lobes in the high cognitive function group. Patients with cognitive dysfunction reportedly have significantly lower functional connectivity than healthy participants, particularly in the frontal lobe (Braakman et al., 2013), where age-related brain volume reductions are most pronounced (Tisserand et al., 2002). Patients with AD and bilateral atrophy of the parietal lobes showed a more rapid decline in cognitive function than other patients with AD (Na et al., 2016). In addition, gray matter atrophy in the posterior and medial parietal regions results in a chain reaction of cognitive dysfunction; the parietal lobe is involved in many cognitive functions, including memory, which is the most prominent dysfunction in AD (Buckner et al., 2005; Jack et al., 2010; Jacobs et al., 2012). Furthermore, the temporal and parietal lobes showed the greatest reduction in brain glucose metabolism, associated with cognitive decline in patients with AD (Small et al., 2000). Therefore, we considered that the significant differences in EEG complexity between the two groups in the frontal, parietal, and temporal lobes were congruent with the regions associated with cognitive decline.

Third, we considered the reasons explaining why high cognitive functions were not identified by the power spectrum analysis but only by MSE analysis. It might be that the complexity captured by MSE analysis reflects complex neural interactions among whole-brain regions, which play a crucial role for emerging cognitive functions (Garrett et al., 2013b); meanwhile, the power spectrum merely reflects local neural activity. Therefore, the complexity profile reflects cognitive function with higher sensitivity than the power spectrum profile.

This study has some limitations. In this study, although we captured changes in cognitive function in healthy older populations using EEG complexity, the number of participants was too small to inform clinical application. In addition, the age distributions of the two groups could hardly be identical due to the small sample size. Therefore, further studies with more subjects are needed to investigate the age distribution under the same conditions in both groups. Further, considering the aim of this study was to develop biomarkers to support the diagnosis of dementia of older populations, longitudinal studies are needed to understand the transition from healthy state to MCI and dementia. In the future, we aim to develop a tool to aid in the diagnosis of cognitive decline across a wider age range and a system to predict cognitive decline by measuring cognitive function scores and EEG over longer time periods.

In this study, we identified the time scale- and site-specific profiles of EEG complexity concerning comprehensive cognitive functions. Despite its limitations, this finding could facilitate the development of a tool for assisting the diagnosis of cognitive decline in older adults.

## Data availability statement

The datasets presented in this article are not readily available because informed consent was not included in the declaration regarding the publication of clinical data. Requests to access the datasets should be directed to SN, nobukawa@cs.it-chiba.ac.jp.

## Ethics statement

The studies involving human participants were reviewed and approved by Ethics Committee of Fukui University. The patients/participants provided their written informed consent to participate in this study.

## Author contributions

YI, SN, KM, MK, TY, MH, YT, and TT designed the study. YI and SN analyzed the results, wrote the main manuscript text, and prepared the figures. MK conducted the experiments. All the authors have reviewed the manuscript. All authors contributed to the article and approved the submitted version.

## Funding

This work was supported by JSPS KAKENHI for the Grant-in-Aid for Scientific Research (C) (Grant No. 20K07964) to TT and (Grant No. 18K11450) to TY. This study was partially supported by the JST CREST (Grant No. JPMJCR17A4).

## Conflict of interest

The authors declare that the research was conducted in the absence of any commercial or financial relationships that could be construed as a potential conflict of interest.

## Publisher's note

All claims expressed in this article are solely those of the authors and do not necessarily represent those of their affiliated organizations, or those of the publisher, the editors and the reviewers. Any product that may be evaluated in this article, or claim that may be made by its manufacturer, is not guaranteed or endorsed by the publisher.
